# Efficient parameter generation for constrained models using MCMC

**DOI:** 10.1038/s41598-023-43433-y

**Published:** 2023-09-28

**Authors:** Natalia Kravtsova, Helen M. Chamberlin, Adriana T. Dawes

**Affiliations:** 1 Department of Mathematics, The Ohio State University, Columbus, OH USA; 2 Department of Molecular Genetics, The Ohio State University, Columbus, OH USA

**Keywords:** Applied mathematics, Computational biology and bioinformatics

## Abstract

Mathematical models of complex systems rely on parameter values to produce a desired behavior. As mathematical and computational models increase in complexity, it becomes correspondingly difficult to find parameter values that satisfy system constraints. We propose a Markov Chain Monte Carlo (MCMC) approach for the problem of constrained model parameter generation by designing a Markov chain that efficiently explores a model’s parameter space. We demonstrate the use of our proposed methodology to analyze responses of a newly constructed bistability-constrained model of protein phosphorylation to perturbations in the underlying protein network. Our results suggest that parameter generation for constrained models using MCMC provides powerful tools for modeling-aided analysis of complex natural processes.

## Introduction

Mathematical modeling is an excellent tool to better understand the temporal and spatial dynamics of complex systems. Some of these systems are known to exhibit specific types of behavior, attracting interest from both natural and mathematical sciences. For instance, consider bistability, a key feature of many natural processes studied in physics^1^, chemistry^2^, material science^3^, and biology, over a range of spatial and temporal scales^4–9^. Any mathematical model of a bistable system is constrained by its ability to reproduce a bistable behavior upon simulations, the minimal requirement that such a model needs to satisfy to meaningfully reflect the underlying natural process. This basic requirement, that we term *model constraint* in what follows, must be satisfied by any vector of parameter values used to simulate model equations. Beyond this basic requirement, each parameter vector would typically result in a different output upon model simulation, which reflects the natural diversity in the underlying system with parameter values representing the sources of such diversity^10,11^. Reproducing this diversity *in silico* requires a large number of parameter vectors for subsequent model simulations that typically assess sensitivity to parameter values^12–14^, robustness^15^, variability in model outputs^10,16^, and/or changes in dynamics in response to changing experimental conditions^17^.

The problem of finding parameter values is extensively studied in the case when quantitative experimental data are available to estimate them. The general approach here is to define an optimization problem to minimize a chosen discrepancy between model output and acquired data (see^18^ for the review of these techniques in the context of mathematical modeling in natural sciences). Very frequently, however, experimental observations are qualitative in nature and cannot be used to numerically estimate the parameter values (see^19^ for discussion of the problem in the context of biological sciences and^20^ for a specific example of this issue). Moreover, due to stringent requirements of a model constraint such as bistability, oscillations, or other specific forms of behavior, it is challenging or impossible to analytically derive conditions on parameter vectors for a given model to satisfy the constraint of interest (^21,22^ are examples of analytical derivations for stability and oscillations constraints, respectively, for smaller models of theoretical importance). To find the conditions on the parameters numerically,^23^ proposes a rigorous methodology that restricts the problem to ordinary differential equation models with piecewise-multiaffine derivatives and constraints expressible via temporal logic formulas^24^. The piecewise-linear nature of the model and ability to express constraints in terms of logical operators enable one to determine interval ranges for parameter values, which can then be sampled to generate parameters for model simulations.

For general model types under general constraints, however, the main practical approach for parameter generation in the absence of quantitative experimental data remains to sample parameter values on/with pre-specified grid/ranges followed by checking the model constraint, if necessary^10,13–16^. The check requires simulating model output with the proposed parameter vector, which incurs high computational cost and significantly slows down the parameter search, especially for complex realistic models with high-dimensional parameter space^20,25,26^. This observation suggests that parameter generation for constrained models could be performed in a more efficient way by “learning” the structure of the parameter space as sampling progresses, reducing the number of model evaluations for sample parameter vectors that are unlikely to satisfy model constraints.

**Figure 1:**
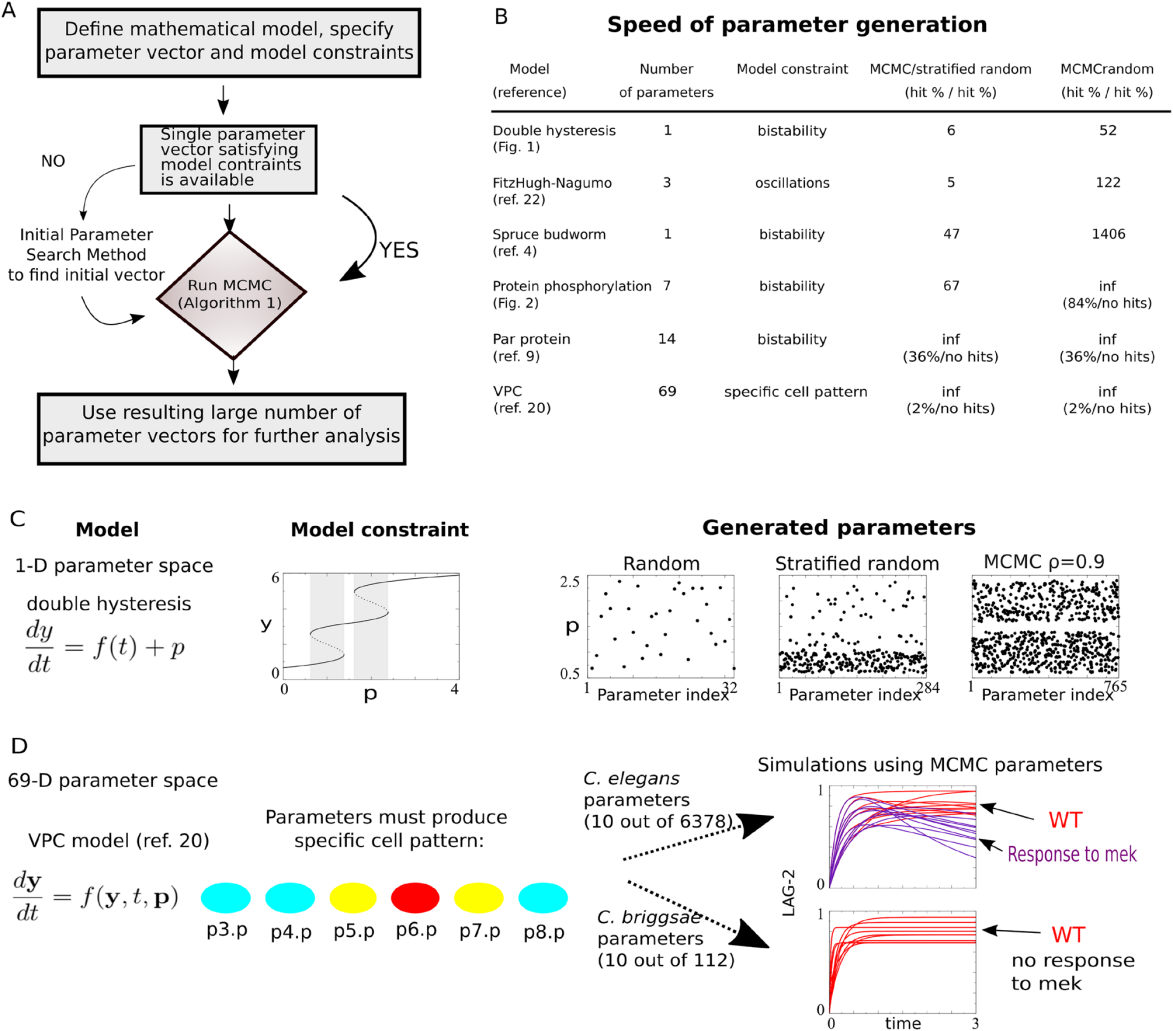
Application of MCMC to constrained model parameter generation. (**A**) Workflow for the proposed MCMC. (**B**) Table comparing the speed of our MCMC to standard parameter search methods for models with varying characteristics. (**C,D**) Analytically tractable double hysteresis model versus analytically intractable VPC model: model structure, constraints, and generated parameters. (**C**) We evaluate our proposed MCMC by applying it to an analytically tractable model, and compare parameter generation methods in terms of speed and accuracy. All methods are executed under the assumption that the parameter space is unknown. (**D**) For the high-dimensional VPC model^20^, other methods fail to identify any working parameter vectors, while our MCMC procedure successfully discovers more than 6000 parameter vectors. This enables the classification of parameters into two distinct classes based on simulation outcomes. Ten sample model simulations, both without and with perturbation (MEK inhibition), from each class are shown, illustrating the successful generation of working parameters for this complex biological model.

**Figure 2:**
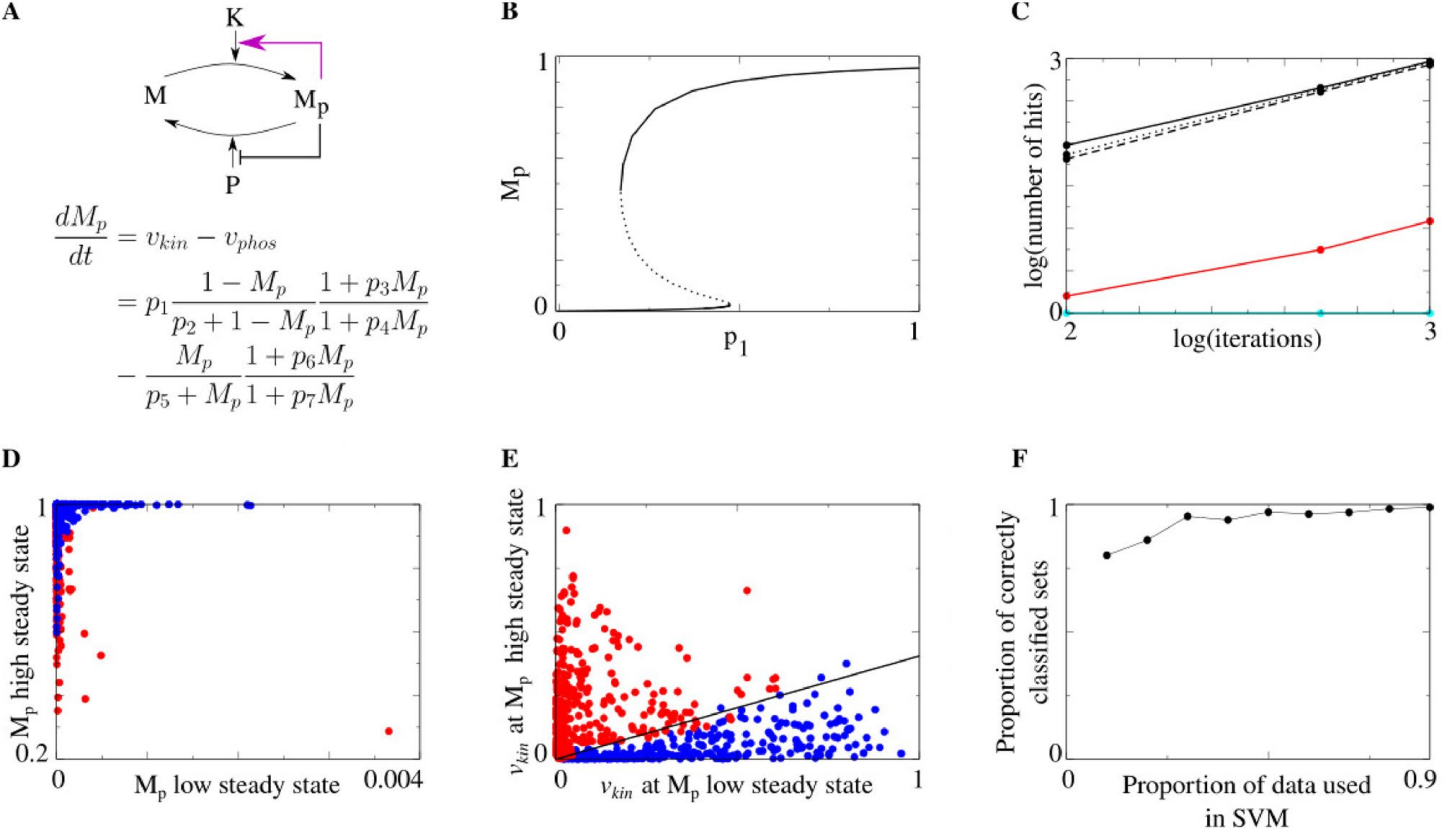
MCMC-generated parameter vectors aid in revealing bistability fingerprint in a high-dimensional model of protein phosphorylation. (**A**) Network diagram and model equations for a generic protein phosphorylation model based on^41^. *M* unphosphorylated protein, $M_p$: phosphorylated protein, *K*: kinase, and *P*: phosphatase. (**B**) Bifurcation diagram for the protein phosphorylation network model with respect to $p_1$, the magnitude of the rate of kinase activity $v_{kin}$ (Solid black line: stable solutions, Black dotted line: unstable solutions). (**C**) Comparison of proposed MCMC, with several choices of $\rho$, the variance scale of the proposal density (Solid black: $\rho =0.1$, Dotted black: $\rho =0.5$, Dashed black: $\rho =0.9$), to stratified random (red) and random (cyan) search methods for a range of iteration numbers. The random search did not find any solutions (defined as 0 on the log scale). (**D**) Steady state values (low vs. high) resulting from simulations of the protein phosphorylation model for each parameter set found by MMH (red: kinase-dependent bistability, blue: kinase-independent bistability). (**E**) Linear separation of kinase-dependent and kinase-independent parameter sets based on in-degree $v_{kin}$ of $M_p$ using support vector machine (SVM). (**F**) Cross-validation analysis of SVM showing the separation is accurate even for small proportions of training data.

We propose a Markov Chain Monte Carlo (MCMC)^27^ approach for the constrained model parameter generation problem by designing a Markov chain that efficiently explores parameter space for a given model (Fig. 1) and demonstrate how generated parameters can be used to obtain and analyze diverse model outputs (Fig. 2). MCMC methods present classical tools to sample from desired target distributions of interest^28^ including recent developments for sampling from distributions with explicitly constrained support such as simplex, sphere, or hypercube^29,30^. In the context of natural processes modeling, MCMC methods have been used extensively for constructing solutions to dynamical systems^31,32^, and inverse problems of parameter estimation from available quantitative experimental data^33^, including data-driven parameter estimation of ordinary^34^ and partial^35^ differential equations. Our settings are different in that experimental data are only available in qualitative form (e.g. Fig. 1D), which allows to formulate the model constraint but prohibits any type of quantitative data fitting and explicit formulation of parameter space constraints. We show that under these settings, the MCMC approach is more efficient than commonly used random sampling methods (Fig. 1B), is capable of handling a wide variety of models with diverse types of constraints (Fig. 1B,C,D), and generates parameter values that lead to insightful conclusions about underlying natural processes (Fig. 2).

In contrast to traditional uses of MCMC^36,37^, the goal of our application is not to produce exact samples from a target distribution, but rather to use MCMC as an exploratory tool that speeds up a parameter search and is able to produce a large number of diverse parameter vectors to be used in subsequent model investigations. To this end, the application of MCMC for parameter generation here does not rely on convergence properties of the underlying chain. In particular, all realizations of the chain are valuable, including the so called “burn-in” period at the beginning, as long as they satisfy model constraints. We propose to use the chain based on the Metropolis-Hastings algorithm (MH)^38–40^ with (in a high-dimensional parameter vector case) component-wise update^27,28,36^. The proposal and target distributions are chosen to satisfy two important requirements for any parameter-generating routine for constrained models: (1) the proposed MCMC generates parameters with high speed in comparison to commonly used alternative procedures, and (2) distant regions of parameter space are explored. Together, properties (1) and (2) allow for the generation of a large number of diverse working parameters for a user-specified constrained model, which would be much more costly or even impossible to generate with more commonly used methods. Our choices for proposal and target distributions, a discussion of our speed maximization procedure, as well as the steps of the algorithm are given in the “Methods” (“Proposed MCMC algorithm”, and Algorithm 1).

We demonstrate the high speed property (1) of the proposed MCMC on a number of models with varying type of constraints, including bistability (models from^4,9^ along with two newly constructed models), emergence of oscillations (model from^22^), and the ability to reproduce a given cell pattern (model from^20^) (Fig. 1B). We observe that the relative gain in speed of parameter generation with MCMC versus two commonly used methods, the random and stratified random parameter search techniques (see “Methods”, “Commonly used methods for parameter generation and their limitations in constrained model case” for their definitions) increases with increasing dimensionality of a parameter space. We further provide three exemplar models with high-dimensional parameter spaces for which our MCMC procedure is the only algorithm able to find working parameters in a reasonable amount of computational time (Fig. 1B).

**Figure 3:**
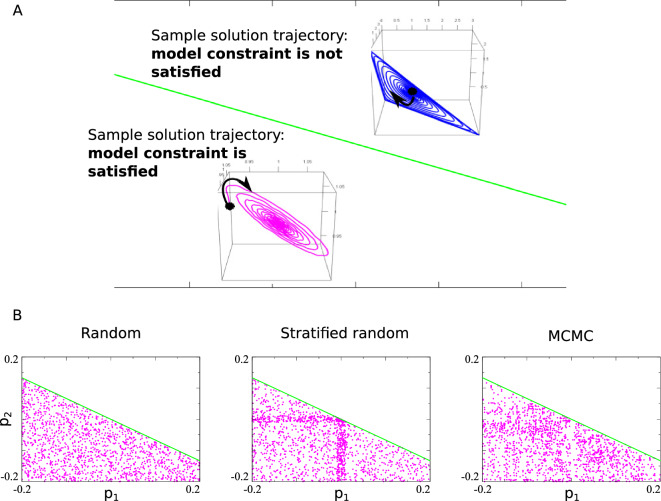
Accuracy of parameter generation from a single run of each algorithm for a Lotka-Volterra model^21^ with parameter space $p \in \mathbb {R}^2$, $0<\Vert p \Vert \ll 1$ (see also Example 4). The steady state (1, 1, 1) is a stable focus provided $p_2 < -\frac{2}{3} p_1$, and we choose this requirement as our model constraint. Parameter vectors satisfying the model constraint lie below the green line. As expected, stratified random sampling is highly dependent on the chosen strata, while MCMC sampling produces uniform coverage comparable to a random sampling scheme.

To illustrate the space-exploring property (2) of the proposed MCMC, we constructed a toy example model with a single parameter and two sequential saddle-node bifurcations (termed *double hysteresis model*) where we theoretically determined that the set of working parameters is exactly the union of two disjoint intervals with known endpoints (Fig. 1C). To test the proposed algorithm, we pretend to be unaware of this information and ask MCMC and two competing methods to find these regions. Our proposed MCMC explores both regions of the parameter space more accurately than a stratified random search by finding parameter values that uniformly cover the desired union of two intervals (Fig. 1C). In addition, the exploration is much more efficient than both the random and stratified random searches (Fig. 1C). Additional examples demonstrating the space exploring-property of our proposed MCMC on analytically tractable models are provided in “Methods”, Fig. 3.

Finally, we demonstrate the use of our proposed methodology to analyze responses of a model of protein phosphorylation to perturbations in the underlying protein network. Based on^41^, we constructed a model of a protein phosphorylation cycle, a universal building block in many cellular signaling pathways^41^, where a protein transitions between phosphorylated and unphosphorylated forms, according to activity of a kinase and phosphatase (Fig. 2A). The model has a seven-dimensional parameter vector, which is required to satisfy a bistability model constraint (Fig. 2B). The bistable behavior of model solutions can be either kinase-dependent or -independent based on the response of the system to perturbation, defined as removing positive feedback to the kinase (magenta arrow in Fig. 2A). The goal is to obtain multiple observations in each of the kinase-dependent/-independent classes in order to identify features specific to each class, where each observation is determined by a parameter set that produces the required behavior upon simulating the model. Figure 2C shows that MCMC is the only alternative that finds a large enough number of different parameter sets for further analysis. Strikingly, this analysis establishes that the classification of kinase-dependent or -independent bistability can be recovered almost perfectly when looking at the rate of kinase activity at steady state in the unperturbed model (Fig. 2E,F), despite no apparent difference in the steady-state levels of the two protein forms (Fig. 2D). This result yields the novel observation that the rate of kinase activity can be used to determine the nature of the bistable mechanism in this protein phosphorylation network.

We designed an MCMC procedure for efficient parameter generation for constrained mathematical models. The procedure uses a properly chosen MH algorithm, allowing for the generation of a large number of parameter vectors for a given constrained model with high speed and large diversity of resulting model outputs in comparison to commonly used routines. Parameter generation for constrained models using MCMC provides a powerful tool for the investigation and analysis of complex high-dimensional models of natural processes.

## Methods

Given a mathematical model that uses constant (and unknown) parameter values, the goal is to find the parameter values that result in a model output with the desired model behavior, which we term satisfying the *model constraint*. Here we consider differential equation models of biological processes, but our algorithm can be applied to any constrained mathematical model that uses constant parameter values. Given a vector of parameter values *p*, consider the map

1 $$\begin{aligned} \phi (p):= {\left\{ \begin{array}{ll} 1 &{} \text { if } p \text { satisfies the model constraint} \\ 0 &{} \text { otherwise } \end{array}\right. } \end{aligned}$$

which indicates whether a given parameter vector satisfies the model constraint.

### Example 1

(*Model constraint illustration: protein phosphorylation model of Fig.* 2A) Consider the model from Fig. 2A with vector of unknown parameter values $p = (p_1 \cdots p_7) \in \mathbb {R}^7_{>0}$

2 $$\begin{aligned} \frac{dM_p}{dt} = p_1\frac{1 - M_p}{p_2+1 - M_p}\frac{1+p_3M_p}{1+p_4 M_p} - \frac{M_p}{p_5+M_p}\frac{1+p_6M_p}{1+p_7M_p} \end{aligned}$$

we are interested in the subset $A \subseteq \mathbb {R}^7_{>0}$ of parameter vectors for which the solution $M_p(t)$ of the dynamical system described by Eq. (2) has two stable and one unstable steady states. This is the bistability model constraint, and parameter vectors $p \in A$ that satisfy this constraint have $\phi (p)=1$, in notation of Eq. (1). All other parameter vector have $\phi (p)=0$. The structure of the set $A \subseteq \mathbb {R}^7_{>0}$ of “good” parameter vectors is unknown, and the goal is to find as many members of *A* as possible.

### Commonly used methods for parameter generation and their limitations in constrained model case

Two most commonly practically used techniques for sampling the parameters for a given mathematical model are uniform random and stratified random searches^10,13^. The comparison results involving these two methods are given in Figs. 1B,C and 2C, and the definitions of these methods are given below.

#### Definition 2

(*Random search*) Given a model with a constant but unknown parameter vector $p \in {\mathbb {R}}^d$, the *random search* procedure proposes a value according to a uniform distribution with bounded support $X \subset \mathbb {R}^d$ specified by the user, i.e. from the distribution with density $f(p) = \frac{1}{\int _X 1 \, dp}\, \chi _{X} (p)$. Given a proposed value for *p*, the model constraints map $\phi (p) \in \{0, 1 \}$ is evaluated for the vector *p*. The next value of *p* is proposed independently of the decision $\phi (p)=0$ or $\phi (p)=1$.

To obtain the results in Fig. 1B, we used the choice $X = [a, b] \times \cdots \times [a, b]$, i.e. the ranges for all coordinates are the same. We chose $a = 0$ and $b = 100$ as plausible ranges for parameter values for a general biological model, except for the FitzHugh–Nagumo model, where the choice of $[-50, 50]$ was used for the 3-parameter vector components that were allowed to be negative (see Eq. 10 in^22^ for more details).

#### Definition 3

(*Stratified random search)* With the same set-up as for the random search, the *stratified random search* proceeds in two steps: for each component of $p \in {\mathbb {R}}^d$, it first randomly selects an interval from $\{[0, 10^{-1}), [10^{-1}, 1), [1, 10), [10, 100] \}$, and then generates a value according to a uniform distribution supported on the chosen interval (making it equally likely to sample from the four regions specified above). Then, the model constraints map $\phi (p) \in \{0, 1 \}$ is evaluated for the vector *p*. The next value of *p* is proposed independently of the decision $\phi (p) \in \{0, 1 \}$.

Note that both methods perform optimally when the user knows the parameter sampling ranges, which is impractical for complex high-dimensional constrained models. Moreover, both methods independently sample parameters at each algorithm iteration, disregarding valuable information about whether the currently proposed parameter vector satisfies the model constraint. These observations suggest the value of a method with “memory”, where one uses information about the current iterate to generate the next iterate, leading us to adopt a Markov Chain Monte Carlo (MCMC) methodology.

### Proposed MCMC for constrained model parameter generation

#### Proposed MCMC algorithm

Given a model, consider a set of constraint-satisfying parameters

$$\begin{aligned} A:=\{p \in \mathbb {R}^d_{\ne 0}: \phi (p)=1 \} \end{aligned}$$

for $d \ge 1$. We remark that frequently the parameter vector *p* represents various rate constants (or combinations of thereof), in which case the common assumption is $p>0$^42^. This is the case for all our empirical evaluations (summarized in Fig. 1B), although the proposed MCMC procedure does not change if parameter values are allowed to be negative (While the case of (some) parameters being exactly zero is of theoretical importance (e.g.^21^), in practice this case leads to redefining a model by omitting the appropriate terms and then assuming the rest of the parameter values are non-zero).

Recall that the structure of the set *A* is unknown, and trial-and-error learning of this structure can be computationally very costly. To sample as many members of *A* as possible, we impose a uniform target distribution on *A* and construct an MCMC chain as follows. The chain starts at a parameter vector $p_0 \in A$ (i.e., the parameter set known to satisfy model constraints) and proposes the next value according to a Gaussian distribution centered at a current value with standard deviation proportional to the current value. This allows the chain to propose small magnitude parameter values if the current value is small in magnitude, i.e. if is there is an evidence that small values satisfy model constraint. If the current value is large in magnitude, the chain is capable of proposing a larger value, since there is evidence that large values satisfy the model constraint. For the $d>1$ case, the update is performed component-wise, which is sometimes referred to as “Metropolis-within-Gibbs” algorithm (Sect. 10.3.3 of^28^) and is widely used in practice for high-dimensional MCMC sampling^27^. The proposal variance is independently scaled for each component, enabling the incorporation of information about how each component acts to satisfy the model constraint.

Formally, the MH chain with states $p=(p(j))_{j=1}^d\in \mathbb {R}^d$ uses the *target* density

3 $$\begin{aligned} \pi (p):= c \cdot \chi _A(p) \end{aligned}$$

where $\chi _A$ denote the indicator function of the set *A* and *c* is the constant that forces $\pi$ to integrate to 1, i.e. $c=\frac{1}{\int \chi _A(p) dp}$ (to ensure that the integral is finite, one needs to assume that *A* is bounded, although the bound can be large in magnitude. This is a reasonable assumption for biologically based models where the underlying parameters are not expected to be extremely large in magnitude). Alternative choices for the target density are discussed in Remark 5. The *proposal* density for proposing a candidate component *y* when at state component *p*(*j*) is

4 $$\begin{aligned} q(p(j),y):= \frac{1}{\sqrt{2 \pi } \rho |p(j)|} e^{-\frac{(y-p(j))^2}{2 \rho ^2 p(j)^2}} \end{aligned}$$

which is of the form of random-walk type of proposals with state-dependent proposal scaling (Sect. 4.3.4 of^36^).

The Metropolis-within-Gibbs (Algorithm A.43 of^28^) updates the *i*th state $p_i=(p_i(j))_{j=1}^d$ componentwise by setting

$$\begin{aligned} p_{i}(j) = {\left\{ \begin{array}{ll} y &{} \text { with probability } \alpha \\ p_{i-1}(j) &{} \text { with probability } 1-\alpha \end{array}\right. } \end{aligned}$$

where

$$\begin{aligned} \alpha := \text {min}\left( \frac{\pi \left( y|p_i(1),\cdots ,p_i(j-1),p_{i-1}(j+1),\cdots ,p_{i-1}(d)\right) \cdot q(y,p_{i-1}(j))}{\pi \left( p_{i-1}(j)|p_i(1),\cdots ,p_i(j-1),p_{i-1}(j+1),\cdots ,p_{i-1}(d) \right) \cdot q(p_{i-1}(j),y)},1 \right) \end{aligned}$$

i.e. the Metropolis-Hastings ratio for the sampling from full conditionals. Observe that for standard implementation of Metropolis-within-Gibbs with the choice $\pi (p)=c \cdot \chi _A(p)$, the ratio reads

$$\begin{aligned} \alpha = {\left\{ \begin{array}{ll} \min \left( \frac{q(y,p_{i-1}(j))}{q(p_{i-1}(j),y)},1 \right) &{} \text { if } \left( p_i(1),\ldots p_i(j-1),y, p_{i-1}(j+1),\ldots , p_{i-1}(d) \right) \in A \\ 0 &{} \text { otherwise } \end{array}\right. } \end{aligned}$$

Computing this ratio requires checking if the vector $\left( p_i(1),\ldots p_i(j-1),y, p_{i-1}(j+1),\ldots , p_{i-1}(d) \right)$ $\in A$ by running an expensive simulation which assesses if the model constraint is satisfied. To avoid this costly evaluation, we propose to ignore the fact that the vector may fail the model constraint and compute the modified ratio

5 $$\begin{aligned} {\widetilde{\alpha }}:= \min \left( \frac{q(y,p_{i-1}(j))}{q(p_{i-1}(j),y)},1 \right) \end{aligned}$$

at each step of Metropolis-within-Gibbs simulation. Only after the whole vector is updated, it is subjected to the model constraint. If the model constraint is satisfied, the updated vector $p_i$ is accepted; if not, the vector is rejected, resulting in the chain returning to the state $p_{i-1}$.

The steps of the algorithm are given in Algorithm 1.
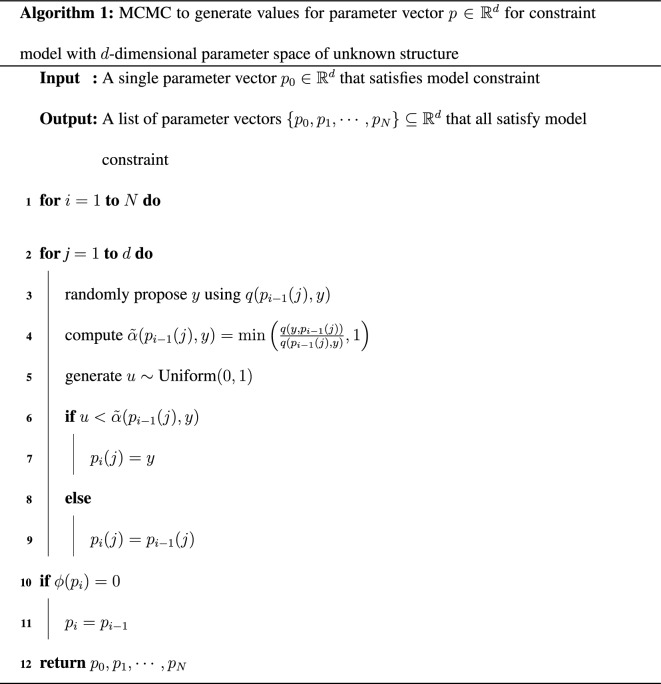


### Speed and accuracy of constrained model parameter generation with proposed MCMC

Note that for the case $d=1$, Algorithm 1 is equivalent to Metropolis-Hastings algorithm (using random-walk type proposal with state-dependent proposal scaling), and for $d>1$, the proposed computational modification to Metropolis-within-Gibbs is to avoid evaluating model constraint until all components of the *d*-dimensional state have been updated. While the update with ${\widetilde{\alpha }}$ may seem wasteful due to possible rejection of the whole newly generated parameter vector after updating all components, the computational cost of the update is very low in comparison to constraint evaluation. Hence, it is cheaper to update all components and possibly lose the updated vector afterwards than carefully check whether earlier updated components work with currently non-updated ones at each component step.

This observation results in significant speed-up of the parameter generation process, illustrated empirically in Fig. 1B. While convergence properties of the chain may alter due to computational speed up described above, our application does not use a target distribution for any probabilistic inference. It is desirable, however, that the proposed MCMC procedure is able to produce diverse enough parameter vectors that uniformly occupy different regions of parameter space, and this property could be demonstrated in cases of simple analytically tractable models. To provide additional to Fig. 1C illustration of this property of chosen MCMC, we consider a slightly more complicated (but still easy to demonstrate graphically) case of $p \in \mathbb {R}^2$ (see Example 4 for model description and Fig. 3 for parameter generation results).

#### Example 4

(*Accuracy of parameter generation tested on analytically tractable model*) Consider Lotka–Volterra model with 3D solutions constructed in^21^ (Eq. (6) in the reference) which uses a parameter vector $p \in \mathbb {R}^2$:

$$\begin{aligned} \frac{d x_1}{dt}&=x_1[(1-x_1)+(1-x_2)+(1-x_3)]\\ \frac{d x_2}{dt}&=x_2[(1-x_1)+(1-x_2)+2(1-x_3)]\\ \frac{d x_3}{dt}&=x_3[(\frac{13}{5}+p_1)(1-x_1)+(\frac{8}{5}+p_2)(1-x_2)+3(1-x_3)] \end{aligned}$$

It is derived analytically (pp. 9–11 of^21^) that the equilibrium point (1, 1, 1) is a stable focus whenever $p_2 < -\frac{2}{3} p_1$, which serves as the model constraint for our example. Similarly to Fig. 1C, for the purpose of testing the parameter generating scheme, we pretend to be unaware of this theoretically determined constraint and subject the model to parameter generation over small parameter ranges $[-0.2, 0.2]$ for each $p_1$ and $p_2$ (these ranges are assumed known, which makes random searches easier than the case of Fig. 1C). As expected, random search covers the constrained parameter region uniformly and stratified random search outputs results highly dependent on the stratification scheme (unknown a priori). MCMC search finds parameter vectors covering the constrained space similar to uniform random search, illustrating the ability of the designed MCMC to produce diverse parameter vector values.

#### Remark 5

(*Practical note on target density*) We observed empirically that for high-dimensional models, the procedure works faster and produces more biologically meaningful results if the target density (Eq. 3) is biased towards smaller magnitude parameter values in the support. In particular, the conditional target density used in Metropolis-within-Gibbs coordinate update using ${\widetilde{\alpha }}$ can be taken

$$\begin{aligned} \pi \left( y|p_i(1),\ldots ,p_i(j-1),p_{i-1}(j+1),\ldots ,p_{i-1}(d)\right) \\ = C \cdot \frac{1}{|y|} \cdot \chi _A(p_i(1),\ldots ,p_i(j-1),y,p_{i-1}(j+1),\ldots ,p_{i-1}(d)) \end{aligned}$$

(where *C* normalizes its integral to 1) resulting in the ratio of posteriors while calculating ${\widetilde{\alpha }}$ in Eq. (5) to be

$$\begin{aligned} \frac{\pi \left( y|p_i(1),\ldots ,p_i(j-1),p_{i-1}(j+1),\ldots ,p_{i-1}(d)\right) }{\pi \left( p_{i-1}(j)|p_i(1),\ldots ,p_i(j-1),p_{i-1}(j+1),\ldots ,p_{i-1}(d)\right) } = \frac{|p_{i-1}(j)|}{|y|} \end{aligned}$$

which changes ${\widetilde{\alpha }}$ to

$$\begin{aligned} {\widetilde{\alpha }} = \min \left( \frac{|p_{i-1}(j)| \cdot q(y,p_{i-1}(j))}{|y| \cdot q(p_{i-1}(j),y)},1 \right) \end{aligned}$$

This choice of target was used when generating parameters for analysis of the protein phosphorylation and VPC models reported in this paper. We note that other choices for the target are possible, and we leave an in depth investigation of their benefits for future work.

### Markov chains for finding initial parameter vectors

All preceding discussion assumed that we have access to a single parameter vector $p_0 \in \mathbb {R}^d$ that satisfies model constraints and hence can be used as a starting value in Algorithm 1. In practice, it is sometimes the case that we don’t have such starting value available. While this case is not the focus of this work, we outline a heuristic approach which we noticed worked well in practice. We call this procedure Initial Parameter Search Method and show it below. We leave the theoretical treatment and rigorous results on practical applications of the method for future study.

For this procedure, we consider, similarly to Eq. (1), relaxed model constraints maps of the form ${\widetilde{\phi }}(p) \in [0, 1]$, where the exact form of ${\widetilde{\phi }}$ is application specific and needs to be specified by a user. The general property is that ${\widetilde{\phi }}$ takes higher values for parameter vectors *p* that produce model outputs closer to the desired output satisfying model constraints, with ${\widetilde{\phi }}(p) = 1$ if model constraints are satisfied. That is,

6 $$\begin{aligned} {\widetilde{\phi }}(p):= {\left\{ \begin{array}{ll} 1 &{} \text { if } \phi (p) = 1\\ 0 &{} \text { if } \phi (p) = 0 \\ t \in (0,1) &{} \text {else} \end{array}\right. } \end{aligned}$$

where permitted values of *t* are those that make ${\widetilde{\phi }}$ well-defined, with higher values of *t* whenever the model output is closer to satisfying model constraints.

We note that such a construction only makes sense in cases where one can assess how close a given model output is to the desired output. An example of the case where such a map is well-defined is the VPC model of^20^, where a parameter vector $p \in \mathbb {R}^{69}$ satisfies model criteria if the model output reproduces a pattern of six cells with cell fates coded by $3 \, 3 \, 2 \, 1 \, 2 \, 3$ (where each code represents certain amounts of proteins of interest). It is known that the pattern with the code value 4 in place of the code value 1 is close to the desired pattern. Thus, we can consider a (well-defined) map ${\widetilde{\phi }}$ as

$$\begin{aligned} {\widetilde{\phi }}(p):= {\left\{ \begin{array}{ll} 1 &{} \text { if } \text { the pattern } 3 \, 3 \, 2 \, 1 \, 2 \, 3 \text { is observed } \\ 0.5 &{} \text { if } \text { the pattern } 3 \, 3 \, 2 \, 4 \, 2 \, 3 \text { is observed } \\ 0 &{} \text { else } \end{array}\right. } \end{aligned}$$

The idea of the Initial Parameter Search Method is to use MCMC which accepts candidate vectors whenever the value of ${\widetilde{\phi }}$ is large enough. In light of the example above, this would mean to accept candidates *p* whenever ${\widetilde{\phi }}(p) \ge 0.5$. For this particular example, we observed in practice that accepting such parameter vectors leads to eventually proposing a candidate $p_0$ with ${\widetilde{\phi }}(p_0) = \phi (p_0) = 1$, i.e. the parameter vector satisfying model criteria that can further be used as starting value for Algorithm 1. We comment that other procedures are possible and are subject to future work. The practical advantage of the current procedure is that the user does not have to switch to running a different code once an initial hit is found.
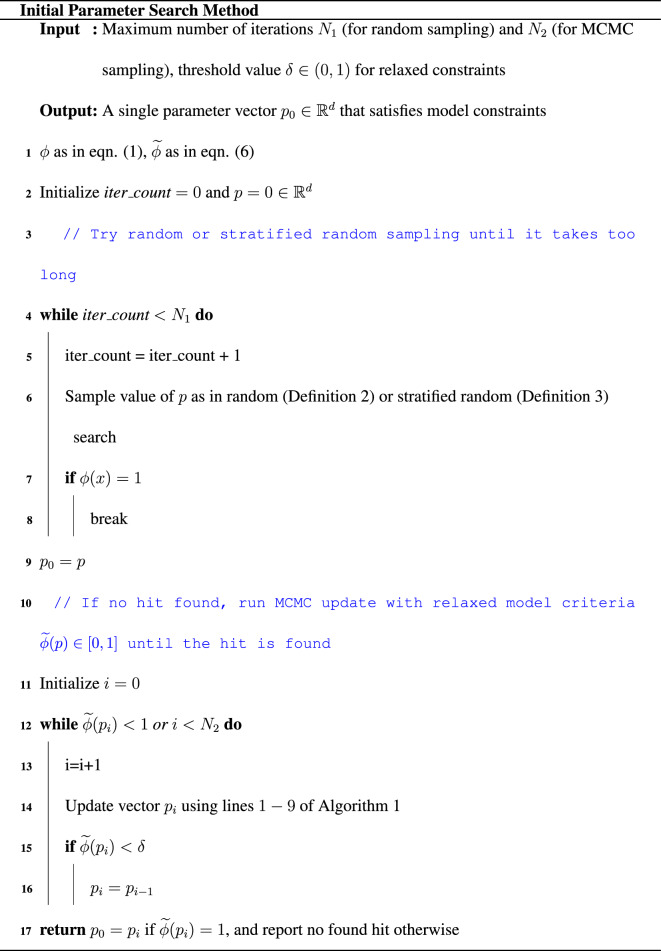


## Data Availability

Any methods, additional references, Nature Research reporting summaries, source data, statements of data availability and associated accession codes are available at https://github.com/adrianadawes/MMH_example.
